# How to Survive a (Juvenile) Piranha Attack: An Integrative Approach to Evaluating Predator Performance

**DOI:** 10.1093/iob/obad032

**Published:** 2023-09-01

**Authors:** A Lowe, M A Kolmann, E W M Paig-Tran

**Affiliations:** Schmid College of Science and Technology, Chapman University, 1 University Dr, Orange, CA 92866,USA; Department of Biology, University of Louisville, Louisville, KY 40292, USA; Department of Biological Science (MH-282), California State University, Fullerton, 800 N State College Blvd, Fullerton, CA 92834-6850, USA

## Abstract

**Figures:**

1. Cory cat panel figure
2. Drawing of bite force measuring equipment and indentation rig
3. *Pygocentrus nattereri* jaw muscle morphology and skull anatomy
4. Box plot grid of number of *Pygocentrus nattereri* bites before puncture along different body regions of *Corydoras trilineatus* during feeding trials results
5. Drawing of color-coded *Corydoras trilineatus* with attack frequencies and average bites until puncture by *Pygocentrus nattereri*
6. Box plot of average voluntary juvenile *Pygocentrus nattereri* bite forces to standard length
7. Panel of linear ordinary least-squares regressions of *Pygocentrus nattereri* bite force to adductor mandibulae mass, standard length, and body mass
8. Ordinary least-squares regressions of voluntary bites to restrained bites of *Pygocentrus nattereri*
9. Panel of indentation tests for intact and removed *Corydoras trilineatus* scutes
10. Panel of indentation tests for *Corydoras trilineatus* body region

**Synopsis:**

There is an evolutionary arms race between predators and prey. In aquatic environments, predatory fishes often use sharp teeth, powerful bites, and/or streamlined bodies to help capture their prey quickly and efficiently. Conversely, prey are often equipped with antipredator adaptations including: scaly armor, sharp spines, and/or toxic secretions. This study focused on the predator–prey interactions between the armored threestripe cory catfish (*Corydoras trilineatus*) and juvenile red-bellied piranha (*Pygocentrus nattereri*). Specifically, we investigated how resistant cory catfish armor is to a range of natural and theoretical piranha bite forces and how often this protection translated to survival from predator attacks by *Corydoras*. We measured the bite force and jaw functional morphology of *P. nattereri*, the puncture resistance of defensive scutes in *C. trilineatus*, and the *in situ* predatory interactions between the two. The adductor mandibulae muscle in juvenile *P. nattereri* is robust and delivers an average bite force of 1.03 N and maximum bite force of 9.71 N, yet its prey, *C. trilineatus*, survived 37% of confirmed bites without any damage. The *C. trilineatus* armor withstood an average of nine bites before puncture by *P. nattereri*. Predation was successful only when piranhas bit unarmored areas of the body, at the opercular opening and at the caudal peduncle. This study used an integrative approach to understand the outcomes of predator–prey interactions by evaluating the link between morphology and feeding behavior. We found that juvenile *P. nattereri* rarely used a maximal bite force and displayed a net predation success rate on par with other adult vertebrates. Conversely, *C. trilineatus* successfully avoided predation by orienting predator attacks toward their resilient, axial armor and behavioral strategies that reduced the predator's ability to bite in less armored regions of the body.

## Introduction

Extensive bony armors were hallmarks of the first great vertebrate radiations, and although the precise function of these armors is contentious (e.g., see Huskey et al. 2020), a defensive role has long been a prominent hypothesis (Romer 1933; Bruet et al. 2008; Meyers et al. 2012; Yang et al. 2012; Huskey et al. 2020; Gai et al. 2022). Biological armors are exceptional for balancing puncture resistance, toughness (energy to fracture), and strength (force to yield) with flexibility arising from the synergism between harder and softer scale components (Bruet et al. 2008; Song et al. 2011; Meyers et al. 2012; Yang et al. 2012, 2013a, 2013b; Zhu et al. 2013; Torres et al. 2015; Sherman et al. 2017; Yang et al. 2019; Lowe et al. 2021). Moreover, bony armors can also play a role in buoyancy control, ion and mineral storage, general hydrodynamics and lift generation, as well as stiffening the skin during swimming (Halstead 1973; Ruben and Bennett 1987; Scheyer 2007; Botella and Fariña 2008; Fletcher et al. 2014; Vernerey and Barthelat 2014). Despite the early appearance of bony armors in vertebrates, armor has been lost, reduced, modified, and regained many times over 500 million years of evolution, making armor just one of the many axes of phenotypic variation defining the most diverse vertebrates, the bony fishes (Sire et al. 2009; Kolmann et al. 2020b; Gai et al. 2022).

**Fig. 1:**
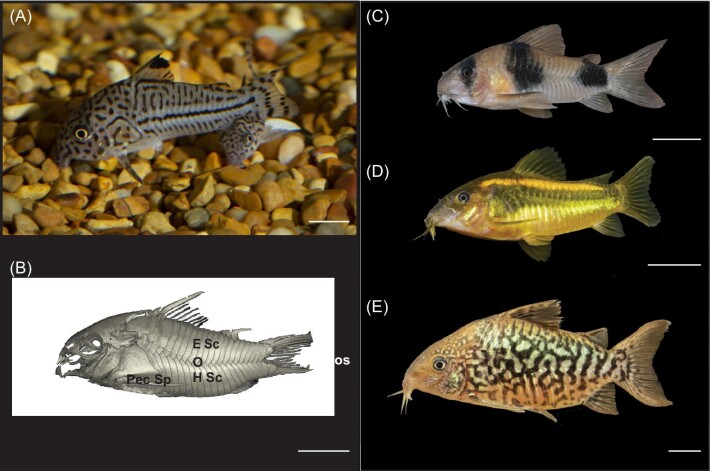
**(A)** Two *Corydoras trilineatus* (threestripe cory) swimming along the substratum in an aquarium. (**B)** Micro CT scan illustrating the axial scutes (dermal plates) in lateral view. Dorsal and ventral rows overlap along the midline. Anterior scutes overlap approximately 30% of the total area with adjacent posterior scutes (Lowe et al. 2021). Anterior (Ant), posterior (Pos), pectoral spine (Pec Sp), dorsal spine (D Sp), epaxial Scutes (E Sc), overlap of scutes (O), hypaxial scutes (H Sc), and adipose spine (A Sp). Examples of cory catfish diversity (**C)–**(**E**). (**C)***C. weitzmani* (two saddle cory), (**D)***C. cf. aeneus* (bronze cory), **(E)***C. pantanalensis* (Pantanal cory). Scale bars = 1 cm. Image A provided by Matthew Gush, CSUF. Image B provided by Adam Summers, UW. Images **(C)–(E)** provided by Oliver Lucanus.

One group of fishes in which armor has evolved several times are the catfishes (Order: Siluriformes), which account for one-quarter of the world's freshwater fish diversity (Sire 1993; Lundberg et al. 1998; Sire et al. 2009). The threestripe cory catfish, *Corydoras trilineatus*, is a small (2–6 cm SL [standard length]), demersal, “armored catfish” (family: Callichthyidae), that lives in shallow streams and rivers in South America, from the upper Peruvian Amazon to the central Amazon basin in Brazil and Colombia (Burgess 1989; Reis 2003). The armor in this genus runs from the opercle to the caudal peduncle and comprises two rows (dorsal and ventral) of laterally located, partially overlapping scutes/dermal plates (Sire 1993; Lowe et al. 2021) (Fig. 1). These scutes can withstand an average of nearly 2 N (range 0.10–4.97 N) of puncture force before fracture (Lowe et al. 2021). However, *C. trilineatus* armor does not span the entirety of the body, the lateral scutes overlap along the midline while the ventral and caudal surfaces have lines of attachment where scutes are absent (Lowe et al. 2021). The head is mostly reinforced; however, the prominent orbital region and the opercular openings are unarmored (Lowe et al. 2021). The line of scute attachment in the underbelly is rarely exposed when *C. trilineatus* swims near the substratum; however, *Corydoras* are facultative air breathers and travel to the surface periodically to gulp air (Kramer and McClure 1980). During forays to the surface to breathe, the ventral surface of *C. trilineatus* and other *Corydoras* catfishes is vulnerable to predators. Laterally, *Corydoras* are equipped with sharp pectoral spines coated with potent toxins, making them difficult prey for predators to handle, as the spines can become lodged in the buccopharyngeal cavities of even large predators (Greven et al. 2006; Alexandrou et al. 2011). Red-bellied piranhas (*Pygocentrus nattereri*), with their razor-sharp teeth and powerful bites (Machado-Allison 1982; Jégu et al. 2003; Grubich et al. 2012), do not seem deterred by the pectoral spines and reinforced axial armor.

Although the ferocious reputation of piranhas is largely hyperbole, most species are piscivorous, and will pursue *Corydoras* in the wild (Nico and Taphorn 1988; Sazima and Machado 1990; Haddad and Sazima 2010). Carnivorous piranhas have sharp, triangular, serrated teeth that slice through the flesh of prey and interlock in a peg-and-socket mechanism to form a saw-like jaw (Shellis and Berkovitz 1976; Kolmann et al. 2019). Adults of two of the largest piranha species, the black piranha (*Serrasalmus rhombeus*) and the red-bellied piranha (*P. nattereri*), have recorded bite forces well over 50 N, with *S. rhombeus* reaching 320 N in a 368 mm individual and *P. nattereri* reaching 84 N ± 68 for specimens averaging 182 ± 55 mm (Grubich et al. 2012; Huby et al. 2019; Velasco-Hogan and Meyers 2021). However, these bite forces may not be reflective of natural feeding bites as measurements were recorded from specimens under stress (held and restrained by the experimenters out of water).

Voluntary *in vivo* bites from free-swimming specimens are notably less powerful than bites in restrained or tetanically induced (electrically stimulated) individuals, as maximal bite force is irregularly used voluntarily (Hernandez and Motta 1997; Herrel et al. 1999, 2001a, 2001b, 2008; Grubich 2005; Huber et al. 2005; Anderson and Westneat 2007). Voluntary bite measurements are more representative of natural bites because they remove the conditions of extreme stress and/or aggression associated with restrained bite tests and because tetanic tests use stimulation voltages several orders of magnitude greater than the vertebrate neuromuscular system (Huber and Motta 2004; Huber et al. 2005; Okada et al. 2007; Freeman and Lemen 2008). The force with which an animal bites can be affected by additional variables including body temperature, intraspecific interactions, and motivational states (Irschick 2002; Anderson et al. 2008; Freeman and Lemen 2008; Pfeiffenberger and Motta 2012). Nevertheless, red-bellied piranhas use a suite of strategies for hunting beyond maximally biting their prey, such as ambush tactics, hunting in large schools, or biting weak and unprotected areas of the prey's body such as the tail, fins, or eyes (Foxx 1972; Northcote et al. 1986; Winemiller 1989; Sazima and Machado 1990). *Pygocentrus nattereri* have been observed to chase *Corydoras* in the wild (Nico and Taphorn 1988; Sazima and Machado 1990; Prudente et al. 2016; Dagosta and De Pinna 2019). The small gape of *P. nattereri* is large enough to ingest *Corydoras* (Huskey et al. 2020) while avoiding the pectoral spines; however, *Corydoras* armor might protect their body during a targeted piranha bite. Performance tests on the armor of *C. trilineatus* indicate that the armor should fail when bitten by adult red-bellied piranhas, but it is unclear whether this is true for juvenile piranhas since the feeding performance of juvenile piranhas is untested (Huby et al. 2019; Lowe et al. 2021; Velasco-Hogan and Meyers 2021).

Predator and prey performance are frequently examined in isolation; here, we integrate both to evaluate the protective providence of fish armor. Our goal was to assess a predator–prey interaction by integrating morphological measurements with behavioral assays. (1) We measured *in situ*, voluntary bite forces from juvenile piranhas as a measure of feeding performance, (2) estimated how piranha bite forces change over ontogeny by combining our data with published estimates, (3) evaluated the variability of prey defenses by using indentation tests on *Corydoras* armor, and (4) characterized predator–prey interactions using *in vivo* videos of piranhas attacking *Corydoras* catfishes. We predicted that unrestrained bite force measurements would be significantly lower than estimated or measured maximal bite forces. Moreover, we expect that these voluntary bite force measurements would better reflect the rate of predator success in natural settings in two ways: (1) the piranha's inability to pierce *Corydoras* armor (*ex vivo*) and (2) the high tendency of live *Corydoras* to escape piranha attacks unscathed.

## Methods

Twelve live, red-bellied piranhas (*P. nattereri*) (8.6–11.3 cm) were acquired via AquascapeOnline.com: Belleville, NJ, USA and imported under a restricted species permit from the California Department of Fish and Wildlife (CDFW, permit number 2485). Piranhas were maintained in 151 L (40 gallons) aquarium breeder tanks (dimensions 92.7 × 48.9 × 43.5 cm) partitioned in half by acrylic boards so that there was one piranha per section, two fish per tank. The acrylic boards had approximately 200 6.35-mm-diameter (0.25 inch) holes cut using a laser cutter (Full Spectrum Laser: Chicago, IL, USA, Hobby Series 20x12 5th Generation CO_2_ Laser Cutter) to allow for water circulation between the two sections. The water was filtered using a freshwater filtration system (Marineland: Blacksburg, VA, USA, ML90750 Magniflow Canister 220 Filter), kept at 24–27°C using two Aqueon 100-Watt heaters per tank, and under a natural photoperiod (13 h light: 11 h dark including natural light from the outside). The piranhas were fed one to two times daily (primarily between 10 and 11 AM and secondarily between 4 and 7 PM) with a mixed diet composed of fresh food appropriate for *P. nattereri* (fish, Omega One: Blacksburg, VA, USA, dried bloodworms, Omega One© and Tetra©: Blacksburg, VA, USA, dried shrimp, raw shrimp, and peas) until satiation. This frequency of feeding was chosen because this regimen has been shown to be sufficient to induce growth in a close relative of *P. nattereri*, the Paraná River pacu (*Piaractus mesopotamicus*) in captivity (Volkoff et al. 2016).

Following live feeding trials and bite force measurements, piranhas were euthanized by submersion in neutrally buffered MS-222 (tricaine methanesulfonate) of at least 98% purity (1% of body weight—lethal dosage: 300–400 ppm). Immediately after euthanasia, the mass and SL of the *P. nattereri* were measured. Their teeth and muscles were then extracted for use in indentation tests and morphometric analysis. All experiments with live *P. nattereri* complied with the guidelines of the California State University, Fullerton Institutional Animal Care and Use Committee (protocol number: 17-R-03).

The 22 *C. trilineatus* used for this study (2.1–3.4 cm SL) were obtained from pet stores in Orange County, California, and online at PetSolutions.com and maintained in one 151 L (40-gallon) aquarium breeder tank using a freshwater aquarium filtration system. The *C. trilineatus* were fed daily with food appropriate for bottom-feeding aquarium fish (dry shrimp pellets from API Fishcare: Chalfont, PA, USA). The water was kept at 24–27°C using two Aqueon: Franklin, WI, USA, 100 W heaters per tank, and at a pH between 7.0 and 8.0 using Aqueon Water Conditioner and API pH Down, conditions like the natural habitat of *C. trilineatus* and other cohabitating *Corydoras* (Tencatt and Ohara 2016; Riley et al. 2019).

### Predator–prey interactions

A total of 12 *C. trilineatus* were used for feeding trials. It was essential to film attacks on live *C. trilineatus* because our piranhas did not attack euthanized specimens (*n* = 2). Live trials were essential for evaluating the defensive posturing used by *C. trilineatus.* For example, to determine if *C. trilineatus* orient themselves so their most heavily armored portion of their body (the dorsal surface) is directed toward the attacker or whether their scutes can be repositioned to slide over one another to mechanically reinforce their armor (Lowe et al. 2021).

Prior to predation trials, *P. nattereri* were acclimated to their tanks for at least 30 days to ensure that they fed normally and were acclimated to the experimenter's presence and had reduced stress levels caused by introduction into a new environment. Each *P. nattereri* individual was starved for 24–48 h prior to the feeding trial, to ensure the fish was hungry and motivated to feed. The water level was lowered by 30% of the tank volume, plants were removed from the tank, and two acrylic boards and an 203.2 × 279.4 mm (8.5 × 11 inch) grid with 12.7 × 12.7 mm (0.5 × 0.5 inch) squares were set up in the tank to reduce the area for the event down to approximately a quarter of the tank (38 L). This created an arena that could be recorded without adjusting the position of the camera, increased the likelihood of catching the event on camera in high definition, and allowed for better analysis. The *P. nattereri* was allowed to acclimate to this changed environment for several minutes before the trial. Each trial began by placing one *C. trilineatus* into a section of the aquarium containing a single piranha. To ensure a defensive response was elicited from exposure to a novel predator and no behavioral learning occurred, no individual *C. trilineatus* was exposed to a piranha more than once.

Trials were video recorded using an Edgertronic SC1 high-speed video camera (San Jose, CA, USA) and a Panasonic DMC-FZ300 to capture anterior and superior views, respectively, of the event. The DMC-FZ300 provided backup in case the Edgertronic SC1 ran out of recording time, as it could only record for 50 s at a time at the settings being used, while the DMC-FZ300 could record for over 3 min. Both cameras were set to record at 240 frames per second (480 p resolution), the maximum frame rate for the DMC-FZ300. The DMC-FZ300 was set to record first, and when an attack appeared imminent, the Edgertonic SC1 was triggered to record, for a maximum of 50 s.

Trials concluded when: (1) the piranha released the deceased *Corydoras* body from its mouth and displayed signs of satiation which included one or more of the following behaviors: cessation of biting, slow swimming movement, and/or lateral undulation of the head or (2) 10 min had passed from the start of the feeding trial. However, if an individual *C. trilineatus* survived or the piranha did not show signs of an imminent attack within 10 min, the *C. trilineatus* was removed using a dip net and euthanized in neutrally buffered MS-222. The piranhas were returned to their habitat (water and plants were replaced and fed normally) for bite force tests and *C. trilineatus* were examined for bite penetration when a *C. trilineatus* body was recovered.

Finally, using QuickTime Player 10.5, each predation trial was scored for the number of bites per strike, number of bites until puncture, attack orientation (0º was the tail region and rotating clockwise so 270º is orthogonal to the right), and the bite location. In addition, we recorded *C. trilineatus* behaviors including orientation, location within the tank, and any notable defensive behaviors including swimming away, turning, bending, anchoring, rotating, and locking the pectoral fins.

### Piranha bite force measurements

Eight of the 12 piranhas were used for voluntary *in vivo* bite force measurements, as the remaining four did not respond to behavioral training for bite force measurements as they did not bite the force plate. We deviated from previous bite force measurements (Huber and Motta 2004; Huber et al. 2005, 2006; Mara et al. 2010) that relied on testing restrained animals or bite forces artificially induced via an electrical stimulus (Huber et al. 2005; Mara et al. 2010; Gidmark et al. 2013). Instead, our experiments required training fish to voluntarily bite a force plate laden with a tasty treat.

**Fig. 2:**
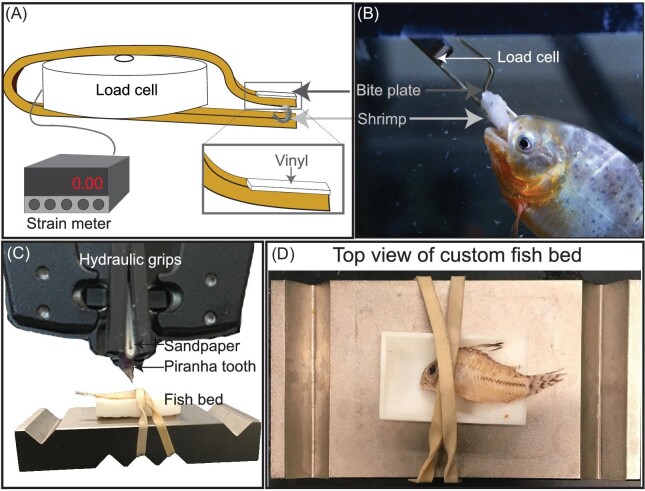
Testing apparatuses. **(A)** Drawing of vinyl wrapped bite plate attached to a load cell connected to a high-speed strain meter. Inset is a drawing of the vinyl covering the brass bite plate. **(B)** Image of *Pygocentrus nattereri* biting the bite plate. **(C)** Instron materials tester hydraulic grips holding piranha teeth for puncturing. **(D)** The customized “fish bed” used to hold the whole fish for puncture tests of intact scutes.

We measured the voluntary bite force of 10 juvenile *P. nattereri* using a customized force gauge consisting of an Omega LCGD-250 low profile miniature load cell (range = 0–113.398 kg) (CT, USA) sealed with aquarium silicone sealant and connected to a digital high-speed load/strain meter (DP-41S, www.omega.com, measuring in MHz) (Fig. 2A), similar to what Grubich et al. (2012) used for black piranhas (*S. rhombeus*). The load cell was equipped with a custom designed brass bite plate, with the ends of the plate serving as the bite surface and the top beam of the brass plate resting against the protruding dimple of the top of the load cell (Fig. 2A and B). The bite surface was covered with vinyl fabric (Joann Fabric and Craft Store, Fullerton, CA, USA) to soften the bite surface and reduce injury or tooth breakage (Lappin and Jones 2014). Voluntary bite force tests complied with the California State University, Fullerton Institutional Animal Care and Use Committee (protocol number: 17-R-03). The DP-41S and load cell with the bite plate were calibrated at three positions across the (top) lever arm of the bite plate using an Instron 5942 mechanical testing machine (Instron Corp., Norwich, MA, USA) to produce a given load. Multiple positions across the bite plate lever arm were calibrated so that the position of the bite plate could be adjusted to better match the gape of each piranha and the size of the shrimp held between the arms of the bite plate to entice an attack (Fig. 2A). Finally, we compared our voluntary bite force values to the restrained bite force values from a previously published study (Huby et al. 2019).

Videos were recorded using both the Edgertronic SC1 and the Panasonic DMC-FZ300 cameras. Two cameras were used as the Edgertronic focused on the bite and Panasonic DMC-FZ300 filmed both the piranha and strain meter from afar. This allowed us to match specific bites to specific strain meter readings. The strain meter and load cell were calibrated to convert from MHz to pounds force (the only two displays the strain meter was capable of) and then into Newtons in calculations afterwards. The reading from the strain meter immediately before the bite and the reading during the bite(s) were recorded and used for calculations. The difference between the two was then converted from pounds force to Newtons. Habituation to the experimenter, the bite plate with shrimp, the light used during filming, and the cameras were performed prior to and during bite force trials. Trials were performed once per week for each piranha over a 4-wk period (four trials per fish) to avoid overfeeding.

### Anatomy and muscle morphology

**Fig. 3:**
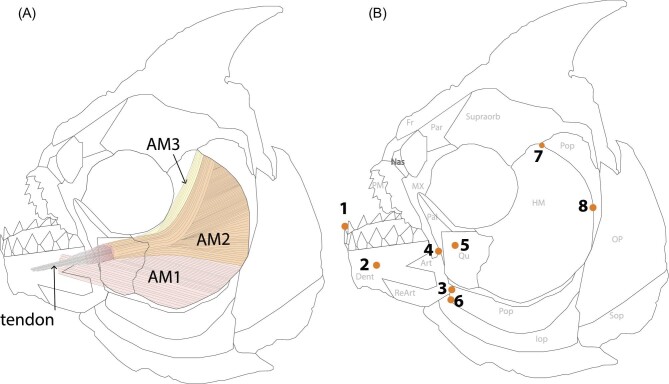
**(A)** Jaw muscle morphology of *Pygocentrus nattereri* and **(B)** skull anatomy. The muscles in **(A)** are subdivisions (AM1, AM2, and AM3) of the adductor mandibulae, the main muscle involved in closing the jaw. The striations for each muscle are shown, with AM2 and AM3 fusing medially into a tendon that inserts on the medial surface of the dentary (shown as if it was lateral). AM1 also inserts on the dorsal-medial surface of the dentary, but is shown to insert laterally in the drawing. The labeled bones in B) are the premaxilla (PM), maxilla (MX), palatine (Pal), dentary (Dent), articular (Art) retroarticular (ReArt), quadrate (Qu), preopercle (Pop), interopercle (Iop), subopercle (Sop), operculum (OP), hyomandibulae (HM), supraorbital (Supraorb), parietal (Par), frontal (Fr), and nasal (Nas). The numbered orange dots represent markers for bite force analysis using MandibLever version 4.0 (4/1/2014): a 2D computational model of jaw biomechanics (Westneat 2003).

Twelve *P. nattereri* individuals were used for anatomical and muscle morphology measurements. Each piranha was euthanized and then weighed (centigrams) and measured for SL (millimeters). Each side of the face was analyzed for morphological comparisons. The piranha jaw muscles consist of the adductor mandibulae (AM) complex composed of three parts—AM1, AM2, and AM3 (Alexander 1964; Fig. 3A). AM1 originates on the ventral portion of the preopercle and wraps around the coronoid process of the articular to insert onto the dorso–medial surface of the dentary (Grubich et al. 2012) (Fig. 3A). AM2 spans the entire suspensorium and makes up more than 80% of the AM mass (Grubich et al. 2012) (Fig. 3A). AM2 fuses with the medial AM3 to form a thick tendon that inserts on the Meckelian fossa on the medial dentary (Grubich et al. 2012) (Fig. 3A). Piranha muscle morphology was examined to measure the length (cm), width (cm), and mass (g) of each component of the AM complex (AM1, AM2, and AM3), the point of insertion of AM2 and AM3 on the mandible, the angle of insertion of AM2 and AM3, length of the top and bottom of the lower jaw, the in-lever (distance from the jaw joint to where AM3 inserts onto the mandible), the out-lever (distance from the jaw joint to the distal-most tooth tip), and the cross sectional area of the AM (Fig. 3B).

These morphometric data were then analyzed with MandibLever version 4.0 (4/1/2014): a 2D computational model of jaw biomechanics (Westneat 2003) to calculate a theoretical maximum bite force for the anterior and posterior for each side of the jaw (Westneat 2003; Anderson and Westneat 2007).

### Puncture performance—indentation tests on *Corydoras* armor

*Corydoras trilineatus* specimens were euthanized prior to indentation tests by submersion in buffered MS-222 (tricaine methanesulfonate) of at least 98% purity (1% of body weight—lethal dosage: 300–400 ppm) with water from the aquarium housing the *C. trilineatus*. After euthanasia, the SL of each *C. trilineatus* individual was measured. Scutes from the dorsal and ventral rows were removed from the anterior, middle, and posterior regions of one side of the body (left or right). Herein anterior scutes always refer to scutes 2–6, middle scutes refer to scutes 9–15, and posterior scutes refer to scutes 19–23. Scutes were either removed individually (single scute puncture test) or together as a complex of two to four overlapping scutes (scute overlap puncture tests) (Lowe et al. 2021). *Corydoras* scutes were dissected from the whole animal for tests using the same procedure and parameters as in Lowe et al. (2021).

Detached scutes were placed so that the ends were each on a small piece of double-sided tape on top of a custom 3D-printed cylinder to hold the scute(s) in place. For indentation tests of specimens with intact scutes, specimens were placed in a customized, 3D-printed (Formlabs Form 2 3D Printer, Formlabs Inc., Somerville, MA, USA) “fish bed” designed to hold the specimen in place (Fig. 2C). The “fish bed” matched the fish body's natural curvature to prevent dorso–ventral rotation during indentation testing (Fig. 2D). A rubber band was secured around the fish bed in a dorso–ventral direction to hold the specimen in place and decrease rostral–caudal movement (Fig. 2D).

We created a custom tooth grip for Instron 200 N pneumatic grips by folding and affixing sandpaper (220 grit, 3M Cubitron II) with cyanoacrylate over the external side of the same vinyl fabric material (folded) used for the bite plate (Fig. 2C). Once dried, we punctured a hole through the center of the folded crease and inserted the tooth so that the crown of the tooth fit through the hole while the base (proximal) of the crown was glued to the sandpaper/fabric holder and the sandpaper was held by the grips (Fig. 2C). Indentation tests were performed using a mechanical loading frame (Instron 5942, Instron Corp., Norwich, MA, USA) and a 50 N load cell.

The apex of one tooth was aligned to puncture the estimated geometric centroid of the scute, and then additional punctures were performed along the scute to test for consistency, one dorsal and one ventral to the geometric centroid. Although it is not ecologically realistic to expect that piranha teeth line up with the centroid of the scutes, we chose the centroid because it presumably is the strongest area of the scute. In addition, we chose the geometric centroid so that we could standardize our punctures as much as possible. The punctures were first analyzed as individual tests (each puncture as an independent puncture). The three punctures for each scute were then averaged.

The loading rate for these experiments was 0.005 mm/s for 1.5 mm or until the tooth fully punctured the scute(s), whichever occurred first (Zhu et al. 2013; Lowe et al. 2021). Although this is presumably slower than the closing speed of the jaws of a piranha, we used this rate to stay consistent with previous literature. We measured force and displacement and used these metrics to calculate force to yield (N), stiffness (N/mm), and work to fracture (joules).

### Statistical methods

The maximum voluntary bite force for each individual was regressed against SL, body mass (grams), and AM mass (g) for scaling analyses using ordinary least-squares (OLS) regression. We chose OLS over other regression models, given concerns about autocorrelation of errors in methods like reduced major axis regression (Smith 2009). For comparisons between our bite force dataset and the data obtained from Huby et al. (2019), we removed any entries from the latter that were missing data. We then used OLS regression to look at scaling across the entire range of piranha ontogeny. In order to assess the compatibility of our data with data from Huby et al. (2019), we used analysis of covariance (ANCOVA) on both bite force datasets. In effect, we used ANCOVA as a test for the homogeneity of slopes between the two datasets, with data from our study and from Huby's classified as a categorical “dummy” variable (Kolmann et al. 2015). This technique was used to determine if slopes of these two datasets were significantly different and therefore, not suitable to analyze together. All data were log_10_ transformed.

For indentation tests, residuals were tested for normality of the puncture data using the Shapiro–Wilk test and for variance using the Fligner–Killeen test, of which they were not normal nor homogeneic for variance. Thus, Kruskal–Wallis tests were applied followed by *post hoc* Nemenyi tests to detect differences in the force to yield, stiffness, and work to fracture among each region of the body and type of scute punctured (single, overlapping detached, and intact) (Zar 2010). Significance was determined at *P*-values <0.05. Because multiple punctures were performed per scute and averaged per scute, statistical analysis tests were performed on both the averaged data set and as individual punctures (not averaged). There were no significant differences found between the two data sets. The analysis of the averaged data is shown. All statistical analyses were performed in R version 4.2.0 (R Core Team 2022) and figures were produced using the package ggplot2 (Wickham 2009).

## Results

### Predator–prey interactions

**Fig. 4:**
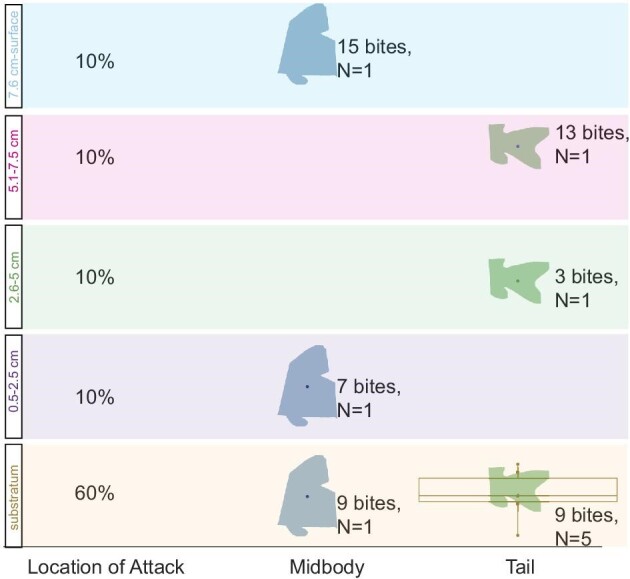
A box plot grid of number of *Pygocentrus nattereri* bites before puncture of *Corydoras trilineatus* during feeding trials based on vertical location in aquaria when *P. nattereri* targeted the mid body versus the tail region. When *Corydoras* were located along the substratum, predation events were more likely to be targeted at the tail. *Corydoras* were subjected to more repeated bites when located in the upper water column. The only regions punctured were the mid body and the posterior regions. The regions of the body are color coded as middle (blue) and posterior (tail). The upper, middle, and lower lines of the boxes represent Q3, median, and Q1 values, respectively. The tail on the substratum was the only subgroup that had a sample size larger than one.

**Fig. 5:**
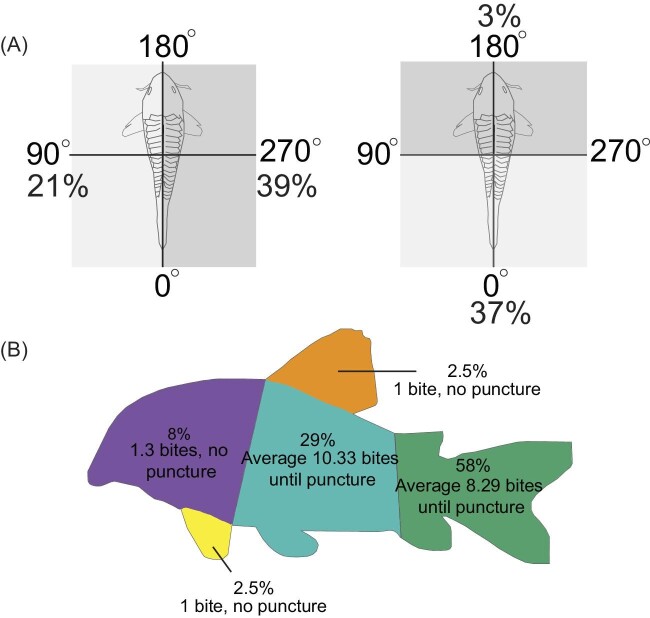
**(A)** Attack frequencies by *Pygocentrus nattereri* on *Corydoras trilineatus* based on direction of predation attack in relation to prey orientation. **(B)** Attack frequencies and average bites until puncture (attacks without bites not included) by *P. nattereri* on a drawing of *C. trilineatus* based on body region, coded by color. The regions of the body were the head (purple), the pectoral fin (yellow), the mid body (blue), the dorsal fin (orange), and the posterior and tail (green).

 We recorded 38 total interactions between *P. nattereri* and *C. trilineatus*. In 14 (37%) of these interactions, no bite landed on *C. trilineatus*. In another 14 of the 38 events (37%) *Corydoras* were bitten (average 1.43 ± 0.85 SD bites per interaction), but the armor was not punctured. Finally, we recorded 10 interactions (26%) that resulted in one or more punctures to *C. trilineatus* armor (*n* = 10 *C. trilineatus*; average of 9.30 ± SD 3.97 bites prior to puncture) (Figs. 4 and 5). Fifty-eight percentage (58%; 22 out of 38 attacks) of the attacks were directed at the tail (Figs. 4 and 5). Seventy percentage of all successful punctures occurred at the tail (7 of 10; Fig. 4). The tail withstood an average of 8.29 bites (±3.73 SD) until failure (Fig. 5B). The other three punctures were located along the mid body, twice directly behind the operculum and one just anterior to the tail (Fig. 4). The mid body was attacked 29% of the time (11 out of 38 times) and withstood an average of 10.33 bites (±4.16 SD) prior to puncture (Figs. 4 and 5). The head was attacked 8% of the time (3 out of 38 times) for an average of 1.3 bites (±0.58 SD), but no attacks were successful in puncturing here. Successful attacks on *C. trilineatus* occurred primarily when *C. trilineatus* were located near the substratum (6 of 10 of successful punctures; Fig. 4). Twenty-five attacks were on the substratum, making the success rate there for *P. nattereri* 24% (6 of 25; Fig. S1A). Four of thirteen (31%) attacks above the substratum resulted in puncture (Fig. S1A). Sixty percentage (23 of 38; 60%) of attacks were orthogonal (perpendicular to the right or left; 15 times to the right, 8 times to the left), and an additional 37% (14 of 38) were from behind at the tail (0º) (Fig. 5A). Defensive strategies employed (multiple strategies can be used in one attack) by *C. trilineatus* included evasive behaviors such as swimming away 87% (33 of 38), rotating the body so that the lateral armor faces toward the attack 18% (7 of 38), and abducting the pectoral fins to expose the venomous spines 16% (6 of 38) (Fig. S1A).

### Bite force

**Fig. 6:**
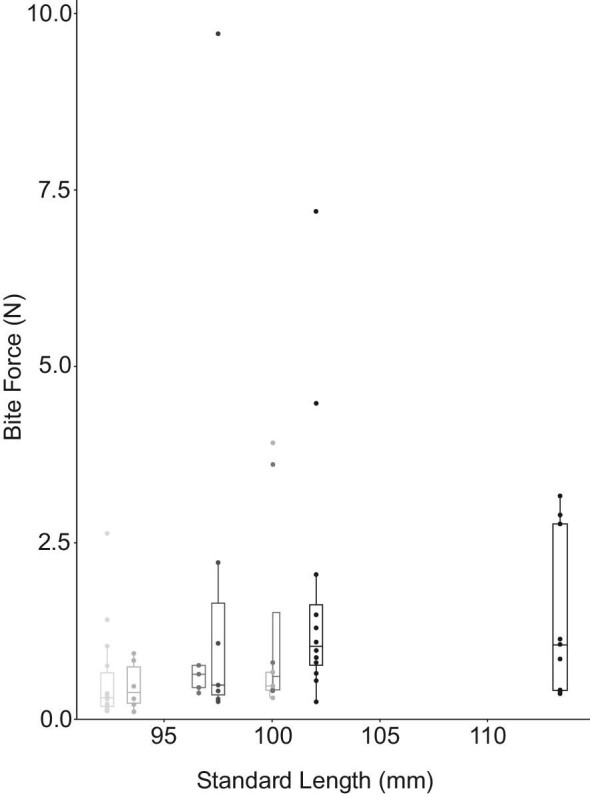
Box plots of voluntary bite forces in Newtons of individual juvenile *Pygocentrus nattereri* measured by standard length (mm). The upper, middle, and lower lines of the box represent third quantile, median, and first quantile values, respectively. Dots represent each trial.

**Fig. 7:**
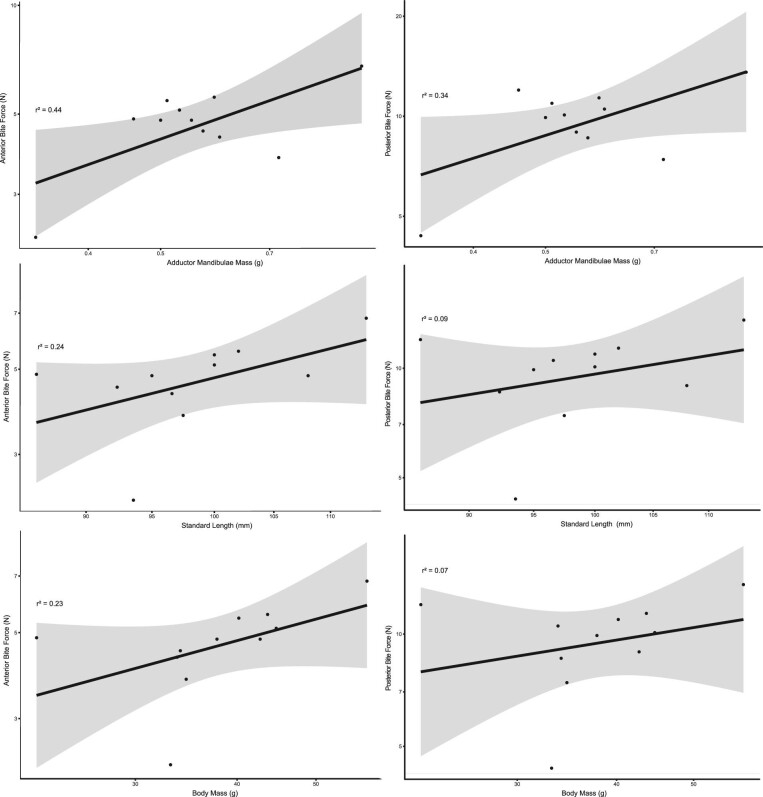
Ordinary least-squares regressions of anterior and posterior bite forces with jaw adductor mass and body size (body mass and standard length; SL). Anterior bite forces represented in the left-hand column, posterior bite forces displayed in the right column. Axes are on a log scale while data are untransformed for clarity of interpretation. Bite forces scaled isometrically with all our variables, although only the regressions with jaw adductor mass (top row) were significant (*P* = 0.02 anterior bite force and 0.05 posterior bite force).

**Fig. 8:**
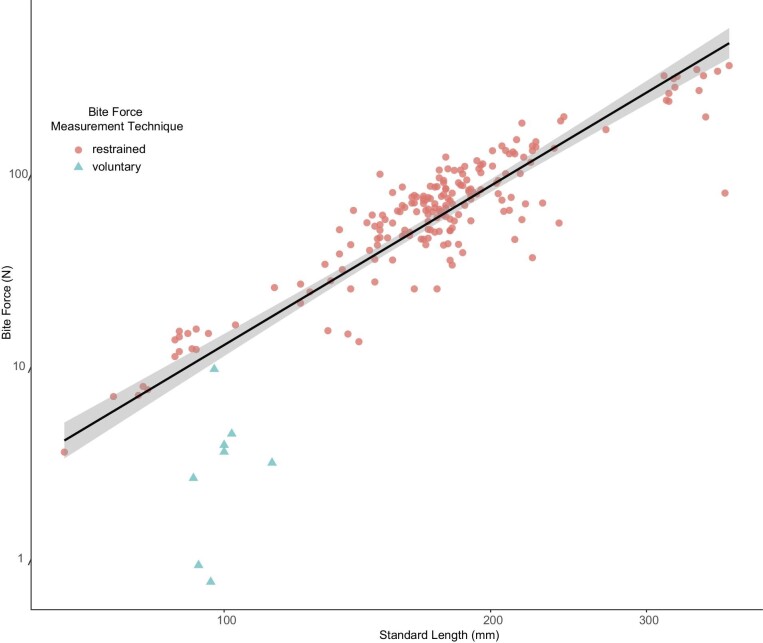
Ordinary least-squares regressions of anterior bite forces and body size (standard length; SL). This study, data are represented by triangles. Huby et al. (2019) are represented by circles. Axes are on a log scale while data are untransformed for clarity of interpretation (*r*^2^ = 0.7577; *P* < 0.001).

*Pygocentrus nattereri* bite forces averaged 1.19 ± 1.68 SD Newtons (*n* = 8) with a maximum voluntary bite force of 9.71 N and a low of 0.10 N (Fig. 6 and Table 1). The maximum bite forces estimated by MandibLever v4.0 averaged 4.75 ± 1.13 SD N for the anterior jaw, 9.79 ± 2.46 SD N for the posterior jaw, and the greatest MandibLever-estimated maximum bite force for any individual was 7.058 N (anterior) and 17.008 N (posterior) (Table 1 and Fig. S1B). The maximum bite forces predicted for each individual were on average 105% (anterior) and 337% (posterior) more than each individual's maximum voluntary bite forces (Table 1); however, one of the piranhas (specimen 4) had a maximum measured bite force (9.71 N) greater than either of the predicted MandibLever v4.0 maximal bite forces for that individual. We report significant positive relationships between the mass of the jaw adductor muscles with anterior and posterior bite force values according to linear regression (*P* = 0.02, 0.05 respectively) (Fig. 7). Similarly, we found positive relationships between bite forces and increasing body size, although these were not significant. Anterior and posterior bite forces scaled isometrically with all the variables; however, only jaw adductor mass was significant (*P* = 0.02). We found a significant interaction when comparing our bite force data methods with those from Huby et al. (2019) suggesting a strong difference between our findings ANOVA*: P* < 0.001) (Fig. 8). However, regression on the combined data sets demonstrated good fit overall (*r*^2^ = 0.7577) and found a significant relationship (*P* = 0.0019) between bite force and SL (slope = 2.75, intercept = −10.1) (Fig. 8).

**Table 1:** Bite force (BF) measurements estimated from MandibLever and measured *in vivo* voluntarily^a^

	Standard length	Body mass (cg)	Anterior max BF	Posterior max BF	Measured voluntary max
ID	(mm)		(N)	(N)	(N)
1	N/A	N/A	1.43	2.82	N/A
2	86	22.16	4.85	11.99	N/A
3	92	34.07	4.49	8.61	2.63
4	97	34.64	3.79	7.41	9.71
5	113	57.76	6.79	13.57	3.17
6	97	33.76	4.32	10.51	0.76
7	94	33.14	2.28	4.37	0.93
8	102	43.61	5.57	11.36	4.48
9	100	45.52	5.13	10.09	3.91
10	108	42.7	4.81	8.96	N/A
11	95	37.79	4.81	9.91	N/A
12	100	40.2	5.45	10.94	3.61

^a^Specimen 1 died before bite tests were performed and mass was not able to be recorded. Specimens 2, 10, and 11 would not voluntarily bite on the force plate.

### Indentation tests

**Fig. 9:**
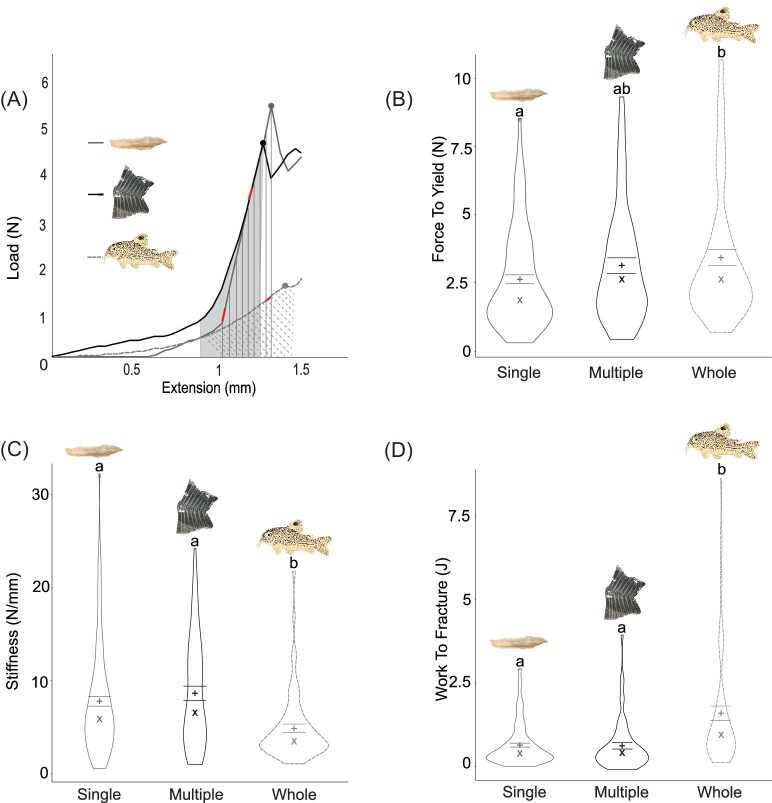
Mechanical indentation tests for scutes intact and removed (single and multiple overlapping) from the *Corydoras trilineatus* body when punctured with a *Pygocentrus nattereri* tooth (*n* = 10). Examples of load–extension curves for each type of scute that was punctured with markings corresponding to the force to yield (dot), stiffness (red line at the linear portion of the curve), and the work to fracture (the area under each curve, marked by the gray shaded region or the corresponding lines). (**B)–**(**D)** Violin plots of the force to yield, stiffness, and work to fracture grouped by type of scute(s) punctured, measured in Newtons, N/mm, and joules, respectively. The width of the curvature of the violin represents the distribution of the data. The bars represent the upper and lower bounds of the standard error. The mean is represented by a plus sign (“+”) and the median is represented by an “X.” Nonmatching letters indicate mean values that differ significantly.

When puncturing with the piranha tooth, the single detached scutes had a significantly lower average ± SE force to yield (2.54 ± 0.18 N) than intact whole body scutes (3.30 ± 0.24 N) (*P* = 0.032), with no significant differences among the other pairs (*n* = 10) (Fig. 9B). The scutes kept intact on the body had significantly lower stiffness (4.91 ± 0.65 N/mm, *n* = 9) than both detached overlapping scutes (7.95 ± 0.48 N/mm, *n* = 9, *P* = 0.00038) and detached single scutes (8.65 ± 0.70 N/mm for detached single, *n* = 9; *P* = 0.00030) (Fig. 9C). Similarly, the intact whole scutes (1.61 ± 0.13 J) required more energy to break than both the detached single scutes (0.77 ± 0.14 J, *P* < 0.0001) and the detached overlapping scutes (0.65 ± 0.10 J, *P* = 0.0029) (Fig. 9D).

**Fig. 10:**
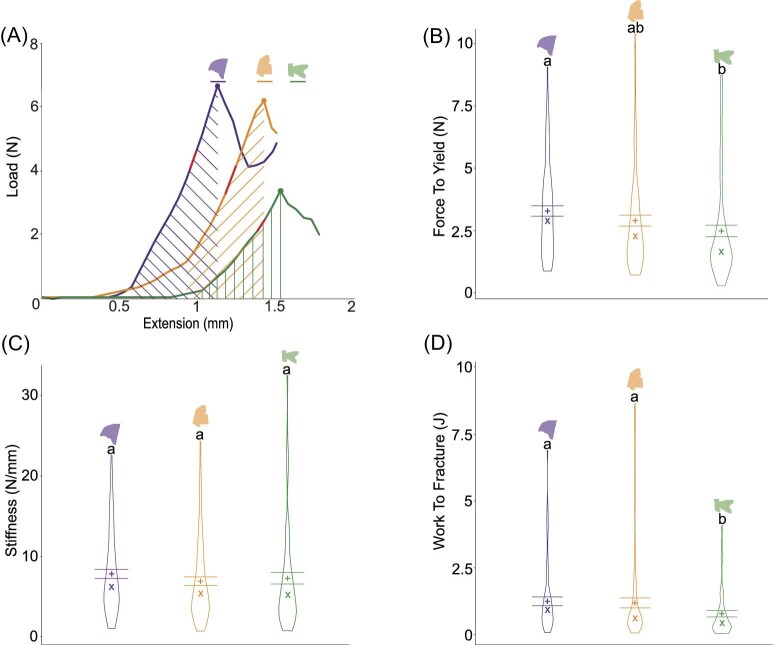
Mechanical indentation tests for different regions of the *Corydoras trilineatus* body when puncturing with a *Pygocentrus nattereri* tooth (*n* = 10). Examples of load–extension curves for each region that was punctured with markings corresponding to the force to yield (colored dot), stiffness (red line at the linear portion of the curve), and the work to fracture (the area under each curve, marked by the corresponding colored lines). **(B)–(D)** Violin plots of the force to yield, stiffness, and work to fracture grouped by region of the body, measured in Newtons, N/mm, and joules, respectively. The regions of the body are color coded as anterior (purple), middle (orange), and posterior (green). The width of the curvature of the violin represents the distribution of the data. The bars represent the upper and lower bounds of the standard error. The mean is represented by a plus sign (“+”) and the median is represented by an “X.” Nonmatching letters indicate mean values that differ significantly.

When puncturing with the piranha tooth, posterior scutes had a significantly lower average ± SE force to yield (2.46 ± 0.22 N) than anterior scutes (3.26 ± 0.23 N) (*P* = 0.0338), with no significant differences among the other pairs (Fig. 10B). There were no significant differences in the stiffnesses of the scutes from each body region (*P* = 0.57) (Fig. 10C). The posterior scutes (0.71 ± 0.13 J) require significantly less energy—approximately 25%—to puncture through than the anterior scutes (1.05 ± 0.13 J) (*P* = 0.0031) and middle scutes (1.04 ± 0.12 J) (*P* = 0.0384) (Fig. 10D).

## Discussion

Most predator attacks fail, and interactions between *Corydoras* catfishes and juvenile piranhas are no exception. But does this mean juvenile piranhas are ineffective relative to other vertebrate predators? *C. trilineatus* individuals were able to survive and escape 74% of *P. nattereri* attacks, putting juvenile piranha predation success rate (26% of all attacks successful) on par with leopards (28%), lions (29%), feral cats (32%), and juvenile toadfish (35%), and more successful than gray reef sharks (5%) (Van Orsdol 1984; Price and Mesinger 1999; Bothma and Coertze 2004; McGregor et al. 2015; Robbins and Renaud 2016). See Vermeij (1982) for additional rates regarding predator success. From the prey's perspective, *C. trilineatus* performs similarly to channel catfish (*Ictalurus punctatus*) which deter and survive (88% survival) predation from largemouth bass (*Micropterus salmoides*) (Bosher et al. 2006). However, predator–prey interactions are extremely complex and predator success is often driven by additional factors including strike distance, substrate characteristics (e.g., rocky or flat), and presence of conspecifics (Pfeiffenberger and Motta 2012). This study took place in an enclosed tank with no form of escape for the prey and no vegetation within the predatory arena for the prey to employ a hiding strategy, likely increasing the predator success rate artificially (Savino and Stein 1989; McGregor et al. 2015). Still, *C. trilineatus* armor withstood an average of 1.43 bites from *P. nattereri* with no damage. The armored scutes of *C. trilineatus* can withstand up to 2.02 N of puncture force (Lowe et al. 2021), which is approximately double the average voluntary bite force of the juvenile *P. nattereri* measured from this study. The armor of *C. trilineatus* does appear strong and tough enough to provide a defense against the bites of a predator, even a persistent predator like piranhas.

Repeated bites appeared to compromise the armor, with an average of nine bites required to puncture through the scutes. Seventy percentage of the successful punctures recorded occurred at the posterior region of the body and/or at the tail region, corresponding to the areas with the mechanically weakest scutes (Lowe et al. 2021). Animal armors must contend with a tradeoff between strength, flexibility, and the weight of the armor (Chen et al. 2012). Thick, bulky armors like turtle shells and adult spearnose poachers (*Agonopsis vulsa:* Family Agonidae) resist puncture but are heavy and limit flexibility because of their rigidity (Achrai and Wagner 2013; Kolmann et al. 2020b). Lighter armors allow for wider ranges of motion, but do not provide as much resistance to puncture (e.g., fish scales near the tail compared to more anteriorly located scales) (Garrano et al. 2012).

*Corydoras* often employ escape responses when facing a predator attack. This quick response may explain why tails were the most common target of attacks. It is possible that piranhas initially target the axial body of the fish but do not anticipate the prey moving away quickly. Or perhaps juvenile piranhas are specifically targeting the tails in anticipation of the prey sensing the attack. This aligns with the *modus operandi* of *P. nattereri* attacks, as this species (and other piranhas) will usually target the tail, other fins, or eyes of its prey (Foxx 1972; Northcote et al. 1986; Sazima and Pombal 1988; Sazima and Machado 1990; Winemiller 1990). Piranhas themselves protect their underbellies with armored keels that are commonly found damaged, suggesting they are vulnerable to ventral and posterior attacks from conspecifics (Sazima and Machado 1990; Kolmann et al. 2020a). In contrast to the “tail-first” strategy used by piranhas, anterior attacks are common for many sharks although not all (Ebert 1991; Heithaus 2001; Michael 2005), summer flounder (Staudinger and Juanes 2010), largemouth bass (Bosher et al. 2006), northern pike (Hoyle and Keast 1988), three-spined sticklebacks (Kjernsmo and Merilaita 2013), and cutthroat trout (Reimchen 1992). The ecological difference between these tail-first or head-first attack strategies may reflect the predator's need to inflict immediate damage to vital organs of the prey, which preempts prey escape, whereas an attack at the tail may reduce the potential for acquiring an injury from prey but could result in the prey escaping (Sargaent and Eberhardt 1975; Strauss and Packer 2013). Posterior attacks are likely a strategy that helps predators avoid injury from prey, such as the sharp teeth, tusks, antlers, etc. from mammals or venomous and sharp pectoral spines from *Corydoras* catfishes (including this study) (Mukherjee and Heitaus 2013; Schendel et al. 2019; Niermann et al. 2020). This tail-first strategy has been documented in some of the most infamous aquatic predators including sharks [white sharks (Long and Jones 1996), Makos (Heitaus 2001), dusky sharks (Dicken et al. 2015), sevengill sharks (Ebert 1991)] and piranhas (Sazima and Pombal 1988; Sazima and Machado 1990). The known tendency of piranhas to feed selectively on prey extremities: tails, paired fins, and scales—might instead speak to the ease of access to these plentiful, comparably safe-to-procure, and replenishable resources (Northcote et al. 1986; Nico and Taphorn 1988; Winemiller 1990).

Armor does not provide an “end-all, be-all” defense—rather, it is one complementary defensive tool among many that gives prey additional chances to survive and seek refuge. We documented several strategies employed by *C. trilineatus* that might help it to survive in the wild. *Corydoras trilineatus* actively prepared for attacks by remaining near the substratum, positioning their body perpendicular to the attack, and then abducting the pectoral fin spines for protection. These sharp, lockable pectoral spines and toxin-secreting axillary glands aid *Corydoras* in weathering attacks by gape-limited predators and deter future attacks by injuring former assailants (Greven et al. 2006). For instance, the pike characin, *Acestrorhynchus pantaneiro*, was documented spitting out a *Corydoras* sp. after attempting to ingest it (Lima and Sazima 2017).

When choosing between consuming the entire prey and risking injury, versus obtaining a tasty morsel consequence-free, piranhas might have found themselves a profitable deal. Juveniles are at an inherent performance disadvantage relative to adults, particularly with respect to bite force, which is largely predicted by body size (Herrel and Gibb, 2006; Anderson et al. 2008). To offset competing with adults, juvenile piranhas (and other juvenile vertebrates in general) typically occupy different niches from adults, with presumably different functional requirements. The average bite performance of juvenile piranhas in this study were anywhere from 8 to 30+ times lower than adults of the same or related species (9.7 N vs. 84 N in *P. nattereri* or 320 N in *S. rhombeus*; Grubich et al. 2012; Huby et al. 2019). This does not mean that juvenile piranhas are ineffective predators, given that their percentage of successful attacks were comparable to adult vertebrates and large terrestrial predators like big cats (Van Orsdol 1984; Price and Mesinger 1999; Bothma and Coertze 2004; McGregor et al. 2015), although our measures are surely biased towards the predator *in vitro*. Most piranhas in their early years behave as ectoparasites rather than outright predators, feeding on parts of prey rather than the whole, in addition to smaller prey like insects and other arthropods (Nico and Taphorn 1988). Our performance measures and behavioral assays strongly suggest that juvenile piranhas are at a performance disadvantage relative to adult piranhas yet mitigate competition with larger individuals and still feed at higher trophic levels by acting as ectoparasites (Sazima and Machado 1990). However, the consequences of interpreting ecological outcomes from different performance metrics can considerably alter our expectations; for example, while bite force typically scales with positive allometry in vertebrates, the bite forces of the juvenile piranhas in this study exhibited negative allometry. However, we note that we measured voluntary bite forces in our experiments and compared these to maximal bite forces measured in adults. The question of how much of a disadvantage juvenile piranhas are at, relative to adults, requires further exploration using diverse morphological, ecological, and behavioral metrics.

Our confidence in experimental measures of predator performance should be based in ecological reality: by evaluating the outcome of predator–prey interactions (Koehl 1996). Some experiments address these issues by comparing static estimates of predator performance to static estimates of prey resilience, for example, by contrasting predator bite forces to prey skeletal failure (Kolmann and Huber 2009; Mara et al. 2010; Pfaller et al. 2011; Grubich et al. 2012; Kolmann et al. 2018). Our study took a combined static and dynamical approach, using voluntary bite tests rather than restrained-specimen testing, direct measures of prey armor resilience, and eco-behavioral outcomes of predator–prey interactions. It is quite likely that because the piranhas in this study were captive bred, there is a difference between them and wild-caught piranhas in several key factors that affect bite force such as jaws, musculature, and lever mechanics (Waddington 1975; Travis 1994; Schlichting and Piggliuci 1998; Erickson et al. 2004). Bite force measurements of restrained individuals have been shown to almost double the voluntary bite forces and are closer to the bite forces elicited from electrical stimulation and those estimated from theoretical models (Huber et al. 2005). However, voluntary bites are more representative of natural feeding behaviors because they avoid being confounded with aggressive or defensive bites (Irschick 2002; Huber and Motta 2004; Huber et al. 2005; Okada et al. 2007; Anderson et al. 2008; Freeman and Lemen 2008). On the flip side, bites on unprotected force transducer surfaces, could underrepresent maximal bite forces (Lappin and Jones 2014). It should be noted that selection likely does not act on voluntary bite force, but rather on maximum capacity because harder bites can give access to more resources in the form of harder, larger seeds for finches (Grant 1981; Grant and Grant 1995; Herrel et al. 2005). Perhaps capturing a range of performance measures relative to prey performance, will give us greater insight into the range of feeding performances regularly employed by vertebrates, encompassing both minimal and maximal estimates of ecologically relevant outcomes (Easterling et al. 2022).

## Conclusions

Defensive armor has evolved several times as a protective mechanism in fishes. This protection, whether it be active (rotation, deflection) or passive (puncture resistance, stress dissipation) is just one multifunctional tool among many that assists armored fishes, like *Corydoras* catfishes, in surviving predator attacks. This study took an integrative approach to understanding the outcomes of predator–prey interactions by evaluating the link between morphology and feeding behavior. Although juvenile piranha bite force is far less potent than that of adults, juvenile feeding performance overall is enough to ensure net predation success as frequently as other adult vertebrates. Our means of measuring feeding performance considers ecological and behavioral norms and may better capture “minimal” performance necessary—an ecologically relevant predictor of predator–prey interactions.

## Supplementary Material

obad032_Supplemental_Files

## Data Availability

The data underlying this article will be shared on reasonable request to the corresponding author. In addition, data from Huby et al. (2019) were provided by Dr. Alessia Huby by permission. Data will be shared on request to the corresponding author with permission of Dr. Huby.
